# Characterization of Recruited Mononuclear Phagocytes following Corneal Chemical Injury

**DOI:** 10.3390/ijms23052574

**Published:** 2022-02-25

**Authors:** Ricardo Lamy, Marie Wolf, Claudia Bispo, Selene M. Clay, Siyu Zheng, Finn Wolfreys, Peipei Pan, Matilda F. Chan

**Affiliations:** 1 Department of Ophthalmology, University of California, San Francisco, CA 94158, USA; ricardo.lamy@ucsf.edu (R.L.); marie.wolf@gladstone.ucsf.edu (M.W.); seleneclay@uchicago.edu (S.M.C.); siyu.zheng@tufts.edu (S.Z.); finn.wolfreys@ucsf.edu (F.W.); peipei.pan@ucsf.edu (P.P.); 2 UCSF Parnassus Flow Cytometry Core Facility, University of California, San Francisco, CA 94143, USA; claudia.bispo@ucsf.edu; 3 Francis I. Proctor Foundation, University of California, San Francisco, CA 94158, USA

**Keywords:** myeloid cells, mononuclear phagocytes, monocytes, macrophages, dendritic cells, corneal injury, MMP12

## Abstract

Mononuclear phagocytes (MP) have central importance in innate immunity, inflammation, and fibrosis. Recruited MPs, such as macrophages, are plastic cells and can switch from an inflammatory to a restorative phenotype during the healing process. However, the role of the MPs in corneal wound healing is not completely understood. The purpose of this study is to characterize the kinetics of recruited MPs and evaluate the role of macrophage metalloelastase (MMP12) in the healing process, using an in vivo corneal chemical injury model. Unwounded and wounded corneas of wild-type (WT) and *Mmp12^−/−^* mice were collected at 1, 3, and 6 days after chemical injury and processed for flow cytometry analysis. Corneal MP phenotype significantly changed over time with recruited Ly6C^high^ (proinflammatory) cells being most abundant at 1 day post-injury. Ly6C^int^ cells were highly expressed at 3 days post-injury and Ly6C^neg^ (patrolling) cells became the predominant cell type at 6 days post-injury. CD11c^+^ dendritic cells were abundant in corneas from *Mmp12^−/−^* mice at 6 days post-injury. These findings show the temporal phenotypic plasticity of recruited MPs and provide valuable insight into the role of the MPs in the corneal repair response, which may help guide the future development of MP-targeted therapies.

## 1. Introduction

The mononuclear phagocyte (MP) system, also called the macrophage system, has central importance in the immune defense of organisms and is comprised of monocytes, macrophages, and dendritic cells [1,2]. Monocytes are the main mononuclear phagocytes in peripheral blood whereas macrophages and dendritic cells are mainly located in tissues. Inflammation is a normal defense response induced by infection or injury [3] and in some cases, it may also lead to tissue fibrosis. The cornea is the transparent tissue in the front part of the eye. The development of corneal fibrosis is associated with tissue opacification, which often leads to visual impairment and blindness. Dysregulated macrophage recruitment and clearance have been shown to be important factors in the development of fibrosis following tissue injury [4,5,6].

Macrophages are crucial for tissue repair and regeneration. In vivo populations of macrophages do not always fit into the traditional classifications of M1 (classically activated macrophages) and M2 (alternatively activated macrophages) defined by in vitro systems [3,7]. Recent studies have shown that macrophages are plastic cells whose phenotype and function can vary depending on the time after injury and that inflammatory monocytes can be converted in situ into macrophages exhibiting a reparative phenotype [8,9,10,11,12]. Thus, the selective reprogramming of macrophages may be a promising therapeutic approach for preventing and treating tissue fibrosis [13].

Differential expression of the cell surface marker Ly6C has been used to identify functionally discrete monocyte/macrophage populations in various tissues. Ly6C^+^ macrophages are thought to be derived from the peripheral circulation and have a pro-inflammatory function [14]. Ly6C^−^ macrophages, on the other hand, are generally regarded as tissue-resident macrophages that play an important role in maintaining immune homeostasis and tissue regeneration [12,14]. Interestingly, in a model of liver injury and regeneration, a Ly6C^lo^ restorative macrophage subset was identified and showed an increased expression of matrix metalloproteinases (MMPs), in particular macrophage metalloelastase (MMP12) [12]. We have previously shown that MMP12 similarly promotes tissue repair in a corneal chemical injury model where MMP12 expression decreased the expression of chemokine CCL2 and reduced the accumulation of macrophages into wounded corneas [15,16]. There are no prior studies assessing MP Ly6C expression over time using an epithelial/stromal chemical injury model of corneal healing. The purpose of this study is to characterize the phenotype and dynamics of recruited MPs in a corneal chemical injury model and evaluate the role of MMP12 in this process. We hypothesized that MP Ly6C expression after corneal injury is highly dynamic and helps to direct the immune and tissue repair response.

## 2. Results

**Figure 1:**
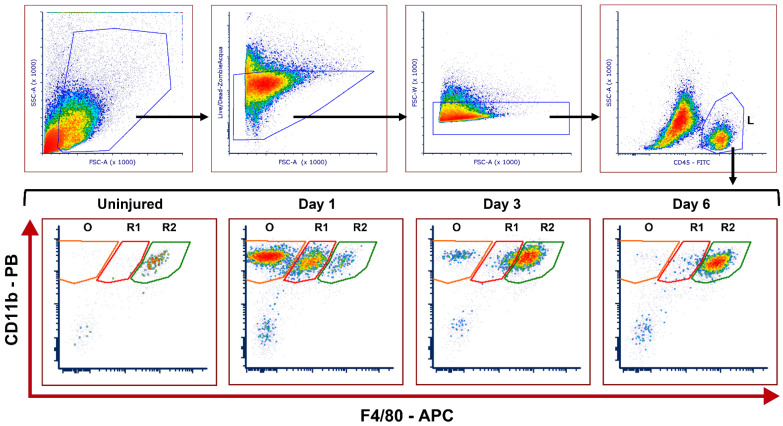
Representative gating strategy of flow cytometry used to identify recruited and resident leukocyte populations and kinetics in uninjured and injured corneas after corneal chemical injury. Corneas from wild type (WT) and *Mmp12^−/−^* (KO) mice at 8–12 weeks old, were collected at 1, 3, and 6 days after an alkaline burn was created by applying filter paper soaked in 0.1 N NaOH. Four corneas from 2 mice were pooled per sample, cut into pieces, and digested with collagenase type I for 1.5 h at 37 °C. Cells were washed with FACS buffer and then incubated for 30 min in a FACS buffer solution containing CD16/32 Fc block. Cells were then washed with FACS buffer and stained for 30 min with the following antibodies: CD45-FITC, CD11B-Pacific Blue, F4/80-APC, Ly-6C-PerCP/Cy5, CD64-PE-Dazzle, CD11c-BrilliantViolet 605, Zombie-Acqua Viability Dye. Cells were washed and then fixed with 2% PFA in PBS overnight (at 4 °C). Cell profiles were acquired on an LSRII flow cytometer (Becton Dickinson, Franklin Lakes, NJ, USA), and were gated based on fluorescence minus one control. The gating strategy is illustrated: Cells → Live → Singlets → CD45^high^ leukocytes (L). Fifty-six corneas were tested in each group (WT and KO); controls (*n* = 3 samples; 12 corneas); day 1 (*n* = 4 samples; 16 corneas); day 3 (*n* = 4 samples, 16 corneas); day 6 (*n* = 3 samples; 12 corneas). CD11b^+^F4/80^high^ resident mononuclear phagocytes (R2) can be seen in the uninjured samples. CD11b^+^F4/80^intermediate^ recruited mononuclear phagocytes (R1) and other CD11b^+^F4/80^−^ leukocytes (O) are seen mainly in day 1 after the chemical injury.

Based on the level of F4/80 expression, three distinct leukocyte (CD45^+^CD11b^+^) subsets were observed in control (uninjured) and injured corneas from WT and *Mmp12^−/−^* mice. A CD11b^+^F4/80^int^ population represented a recruited mononuclear phagocyte population (R1), and a CD11b^+^F4/80^high^ population represented a resident mononuclear phagocyte population (R2) (Figure 1). The CD45^+^ CD11b^+^ F4/80^−^ subset (O) represents a population of other immune cell types that are not part of the MP system, and presumably comprise granulocytes, lymphocytes, and mast cells.

### 2.1. Dynamics of Corneal Mononuclear Phagocytes following Chemical Injury

**Figure 2:**
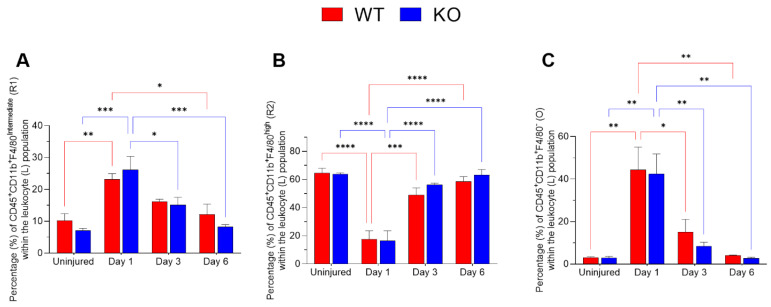
Corneal leukocyte dynamics after chemical injury. Corneas from wild type (WT) and *Mmp12^−/−^* (KO) mice at 8–12 weeks old, collected at 1, 3, and 6 days after an alkaline burn was created by applying filter paper soaked in 0.1 N NaOH. Four corneas from 2 mice were pooled per sample, day 1 (*n* = 4 samples; 16 corneas); day 3 (*n* = 4 samples, 16 corneas); day 6 (*n* = 3 samples; 12 corneas). All data show as mean ± SEM percentage of corneal leukocytes (L). (**A**) The frequency of CD11b^+^F4/80^intermediate^ recruited mononuclear phagocytes (R1) significantly changed over time (two-way ANOVA *p* = 0.0002) and was statistically significantly higher on day 1 after the chemical injury in WT and KO mice in comparison to other time points. (**B**) The percentage of CD11b^+^F4/80^high^ resident mononuclear phagocytes (R2) changed over time (two-way ANOVA *p* < 0.0001) and they were more frequent in uninjured corneas. (**C**) CD45^+^CD11b^+^F4/80^−^ (O) frequency changed over time (two-way ANOVA *p* < 0.0001) with a significantly higher percentage observed on day 1 after injury. The differences between WT and KO leukocyte populations were not statistically significant. Tukey’s multiple comparison-adjusted *p*-value * <0.05; ** <0.01; *** <0.001; **** <0.0001.

The relative proportions of MP subsets following corneal injury were quantified over a period of 6 days (Figure 2). The R2 subset, CD11b^+^F4/80^high^, which represents resident mononuclear phagocytes, was abundant in uninjured corneas from both WT and *Mmp12^−/−^* mice. The relative proportion of R2 was significantly reduced following corneal injury (day 1) and progressively recovered over successive post-injury time points in corneas from WT and *Mmp12^−/−^* mice (two-way ANOVA, *p*-value < 0.001; WT uninjured vs. day 1 adjusted *p*-value < 0.0001; KO uninjured vs. D1-adjusted *p*-value < 0.0001; Figure 2B). The CD11b^+^F4/80^int^ subset (R1) represents a recruited mononuclear phagocyte population. R1 became significantly more abundant one day post-injury in corneas from both WT and *Mmp12^−/−^* mice compared with uninjured corneas (two-way ANOVA, *p*-value < 0.0001; WT uninjured vs. D1 adjusted *p*-value = 0.0075; KO uninjured vs. D1 adjusted *p*-value = 0.0001; Figure 2A).

There was also a significant difference over time in the O subset (two-way ANOVA, *p*-value < 0.0001; WT uninjured vs. D1 adjusted *p*-value = 0.0012; KO uninjured vs. D1 adjusted *p*-value = 0.0019; Figure 2C).

The differences between WT and *Mmp12^−/−^* were not statistically significant for R1, R2 or O subsets (*p* > 0.05).

### 2.2. Ly6C Expression in Recruited Mononuclear Phagocytes after Chemical Corneal Injury

**Figure 3:**
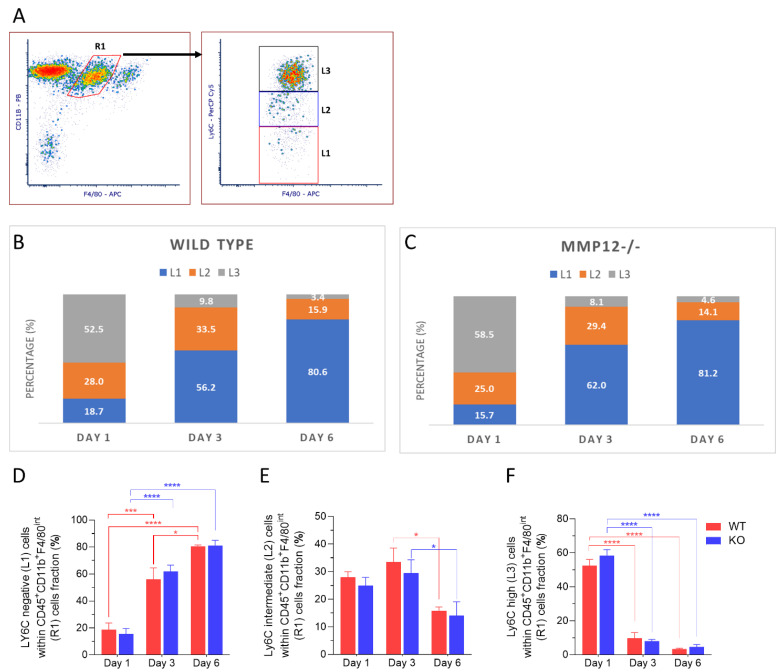
Dynamics of Ly6C expression in recruited mononuclear phagocytes (R1) after corneal chemical injury. Corneas from wild type (WT) and *Mmp12^−/−^* (KO) mice at 8–12 weeks old, collected at 1, 3, and 6 days after an alkaline burn was created by applying filter paper soaked in 0.1 N NaOH. Four corneas from 2 mice were pooled per sample, day 1 (*n* = 4 samples; 16 corneas); day 3 (*n* = 4 samples, 16 corneas); day 6 (*n* = 3 samples; 12 corneas). Cell profiles were acquired on an LSRII flow cytometer (Becton Dickinson, Franklin Lakes, NJ, USA), and were gated based on fluorescence minus one control. All data are shown as the mean ± SEM. (**A**) Based on Ly6C-PerCp-Cy5 staining, CD11b^+^ F4/80-APC ^intermediate^ recruited mononuclear phagocytes (R1) were grouped into 3 groups: Ly6C negative (L1), Ly6C intermediate (L2), Ly6C high (L3). (**B**,**C**) L1, L2, L3 percentage fractions over time in WT and KO mice, and average percentage of each group are presented. (**D**) Average percentage of L1 significantly changed over time (two-way ANOVA, *p*-value < 0.0001) and is higher on day 6 in both WT and KO mice. (**E**) Average percentage of L2 changed over time (two-way ANOVA, *p*-value = 0.0023) and was significantly higher on day 3 than day 6 in both WT and KO mice. (**F**) Average percentage of L3 changed over time (two-way ANOVA, *p* < 0.0001) and was significantly higher on day 1 in both WT and KO mice. The differences between WT and KO MP populations, based on Ly6C expression, were not statistically significant. Tukey’s multiple comparison-adjusted *p*-value * <0.05; *** <0.001; **** <0.0001.

Expression of Ly6C revealed three distinct populations within the R1 subset: Ly6C^neg^ (L1), Ly6C^int^ (L2), and Ly6C^high^ (L3) (Figure 3A). The relative proportions of these populations showed dynamic changes following injury (Figure 3B,C). On day 1 post-injury, the Ly6C^high^ (L3; proinflammatory) subset was predominant, comprising 52.5% in corneas from WT mice and 58.5% in corneas from *Mmp12^−/−^* mice. On days 3 and 6 post-injury, the Ly6C^neg^ (L1) subset was predominant with 56.2% for WT and 62% for *Mmp12^−/−^* at day 3 post-injury and 80.6% for WT and 81.2% for *Mmp12^−/−^* at day 6 post-injury. The percentage of Ly6C^neg^ (L1) cells was significantly increased (*p* < 0.0001) at days 3 and 6 post-injury compared with day 1 in corneas from WT (18.7% versus 56.2% and 80.6%, respectively) and *Mmp12^−/−^* (15.7% versus 62% and 81.2%, respectively) mice (Figure 3D). By contrast, the percentage of Ly6C^high^ (L3) cells was significantly reduced (*p* < 0.0001) at days 3 and 6 post-injury compared with day 1 in corneas from WT (52.5% versus 9.8% and 3.4%, respectively) and *Mmp12^−/−^* (58.5% versus 8.1% and 4.6%, respectively) mice (Figure 3F). The percentage of Ly6C^int^(L2) cells also changed over time (*p* = 0.0032), showing a significant reduction between days 3 and 6 post-injury in corneas from WT (33.5% versus 15.9%) and *Mmp12^−/−^* (29.4% versus 14.1%) mice. These results show a shift in Ly6C expression by MP cells following injury with the Ly-6C^high^ (proinflammatory) subset being expressed early and the Ly6C^neg^ being expressed later at the time points of maximal mononuclear phagocyte recruitment.

### 2.3. CD11c^+^ Dendritic Cells Are Highly Abundant in Corneas from Mmp12^−/−^ Mice 6 Days after Chemical Corneal Injury

**Figure 4:**
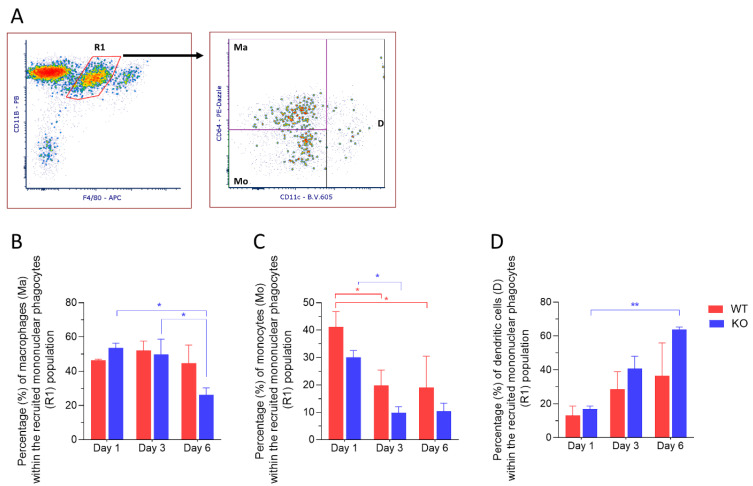
Dynamics of recruited monocytes and dendritic cells after corneal chemical injury. Corneas from wild type (WT) and *Mmp12^−/−^* (KO) mice at 8–12 weeks old, were collected 1,3 and 6 days after an alkaline burn was created by applying filter paper soaked in 0.1 N NaOH. Four corneas from 2 mice were pooled per sample, day 1 (*n* = 4 samples; 16 corneas); day 3 (*n* = 4 samples, 16 corneas); day 6 (*n* = 3 samples; 12 corneas). Cell profiles were acquired on an LSRII flow cytometer (Becton Dickinson, Franklin Lakes, NJ, USA), and were gated based on fluorescence minus one control. All data are shown as the mean ± SEM. (**A**) Representative gating strategy of flow cytometry used to identify dendritic cells (D), macrophages (Ma), and monocytes (Mo) within the recruited mononuclear phagocyte (R1) population. (**B**) Average percentage of CD11b^+^ F4/80 ^intermediate^ CD11c^−^ CD64^+^ macrophages (Ma) was significantly lower in KO mice on day 6 when compared to days 1 and 3. (**C**) Average percentage of CD11b^+^ F4/80 ^intermediate^ CD11c^−^ CD64^−^ monocytes (Mo) was significantly higher on day 1 in both WT and KO mice. (**D**) Average percentage of CD11b^+^ F4/80 ^intermediate^ CD11c^+^ dendritic cells (**D**) was significantly higher in KO mice on day 6 when compared to day 1. Tukey’s multiple comparison-adjusted *p*-value * <0.05; ** <0.01.

Dendritic cells have been found in the normal corneal stroma and play a pivotal role in the initiation of antigen-specific adaptive immune response and in the induction of tolerance [17]. We next sought to characterize the recruited MP population based on staining of CD11c, a marker for dendritic cells and CD64, a marker for macrophages. Three distinct subpopulations were identified: CD11c^+^ dendritic cells (D), CD11c^−^ CD64^−^ recruited monocytes (Mo) and CD11c^−^ CD64^+^ recruited macrophages (Ma) (Figure 4A). CD11c^+^ dendritic cells showed an increase in expression over time after injury and showed a significant increase in expression at 6 days post-injury in corneas from *Mmp12^−/−^* mice compared with 1-day post-injury (16.9% versus 63.9%; *p* = 0.0061) (Figure 4D). These results show that CD11c^+^ dendritic cells are highly expressed in corneas from *MMP12^−/−^* mice 6 days after chemical corneal injury. In contrast, the proportion of CD11c^−^ CD64^+^ recruited macrophages significantly decreased at 6 days post-injury in corneas from *Mmp12^−/−^* mice compared to days 1 or 3 post-injury (53.47% and 49.85% versus 26.23%; *p* = 0.0196 and 0.0438, respectively) (Figure 4B).

The frequency of F4/80^int^CD11c^−^ CD64^−^ recruited monocytes within each group showed a significant reduction from 1 day post-injury to 3 days post-injury in corneas from both WT (41.16% versus 19.82%; *p* = 0.00312) and *Mmp12^−/−^* mice (30.04% versus 9.84%; *p* = 0.0429). No difference was observed when comparing WT vs. *Mmp12^−/−^* on days 1, 3 or 6 (*p* = 0.3872; *p* = 0.4777; *p* = 0.6882, respectively) (Figure 4C).

### 2.4. Ly6C Expression in Recruited Macrophages after Chemical Corneal Injury

**Figure 5:**
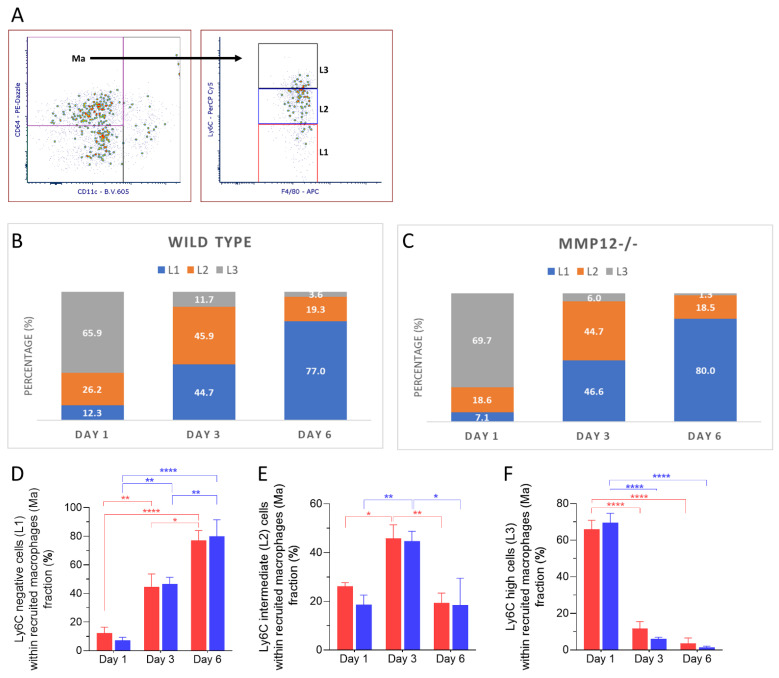
Dynamics of Ly6C expression in recruited macrophages (Ma) after corneal chemical injury. Corneas from wild type (WT) and *Mmp12^−/−^* (KO) mice at 8–12 weeks old were collected at 1,3 and 6 days after an alkaline burn was created by applying filter paper soaked in 0.1 N NaOH. Four corneas from 2 mice were pooled per sample, day 1 (*n* = 4 samples; 16 corneas); day 3 (*n* = 4 samples, 16 corneas); day 6 (*n* = 3 samples; 12 corneas). Cell profiles were acquired on an LSRII flow cytometer (Becton Dickinson, Franklin Lakes, NJ, USA), and were gated based on fluorescence minus one control. All data are shown as the mean ± SEM. (**A**) Based on Ly6C-PerCp-Cy5 staining, CD11b^+^F4/80^int^ CD11c^−^ CD64^+^ recruited macrophages (Ma) were grouped into 3 groups: Ly6C^neg^ (L1), Ly6C^int^ (L2), Ly6C^high^ (L3). (**B**,**C**) L1, L2, L3 percentage fractions over time in WT and KO mice, and average percentage of each group are presented. (**D**) Average percentage of L1 significantly changed over time (two-way ANOVA, *p*-value < 0.0001) and was higher on day 6 in both WT and KO mice. (**E**) Average percentage of L2 changed over time (two-way ANOVA, *p*-value = 0.0002) and was significantly higher on day 3 than on days 1 and 6 in both WT and KO mice. (**F**) Average percentage of L3 changes over time (two-way ANOVA, *p* < 0.0001) and was significantly higher on day 1 in both WT and KO mice. The differences between WT and KO MP populations, based on Ly6C expression, were not statistically significant. Tukey’s multiple comparison-adjusted *p*-value * <0.05; **<0.01; **** <0.0001.

The frequency of Ly6C^neg^ cells within the recruited macrophages population increased over time and was statistically significant on days 3 and 6 in corneas from WT and *Mmp12^−/−^* mice compared to day 1 (Figure 5D). The frequency of Ly6C^int^ recruited macrophages was significantly higher on day 3 (45.9%, 44.7%) than on days 1 (26.2%, 18.6%) or 6 (19.3%, 18.5%) for both WT (day 1 vs. day 3 *p* = 0.0399; day 3 vs. day 6 *p* = 0.0096) and *Mmp12^−/−^* (day 1 vs. day 3 *p* = 0.0061; day 3 vs. day 6 *p* = 0.0106) (Figure 5E). Ly6C^high^ recruited macrophages were significantly more abundant on day 1 in corneas from WT and *MMP12^−/−^* mice (Figure 5F).

## 3. Discussion

In this study, we used flow cytometry to characterize the kinetics of corneal leukocytes, the impact of MMP12 gene knockout, and the phenotype of recruited MPs following corneal chemical injury. The MP system is the main first-line immune defense against pathogens and we found that this population of recruited cells responded acutely following corneal chemical injury, being detected at the highest levels on the first day post-injury and then decreasing steadily over the week following injury. Within the recruited MP population, cells expressing high levels of Ly6C were predominant in the early phase after injury (day 1) while cells expressing intermediate levels of Ly6C increased in frequency on day 3 and cells with low expression of Ly6C were the predominant cell type at 3 and 6 days after injury. These findings show the very dynamic changes in MP Ly6C expression acutely after corneal injury.

MP cells, in particular macrophages, have a key role in tissue repair [1,2]. Many studies have shown that inflammatory monocytes can be converted in situ into cells exhibiting a reparative phenotype [8,10,12]. Our findings show that following corneal chemical injury, Ly6C^int^ restorative MP (CD11B^hi^ F4/80^int^) are mostly highly expressed on day 3 post-injury. Differentiation of corneal MPs based on surface markers is not trivial, and although many studies have considered that all CD11b^+^ F4/80^+^ represent macrophages, recent studies have suggested that CD64 surface marker can help differentiate corneal macrophages from monocytes [18]. Our subsequent analysis of the Ly6C expression gating for CD11b^+^ F4/80^int^ CD11c^−^ CD64^+^ recruited macrophages showed a similar frequency pattern with a gradual shift in frequency from Ly6C^high^ to Ly6C^int^ on day 3 and Ly6C^neg^ on day 6.

Restorative Ly6C^lo^ macrophages expressed during tissue repair and regeneration have been shown to highly express MMP12 [12,19]. Because we have previously shown MMP12 to promote corneal repair [15,16,20], we expanded our study to investigate the kinetics of MP cells not only in WT mice, but also in MMP12 knockout mice. MMP12 is a member of the matrix metalloproteinase (MMP) family and is secreted mainly by macrophages. MMP12 has been shown to be a key factor in regulating the development of fibrosis in several models of tissue injury, including lung and liver injuries [15,21,22,23,24]. In a previous study, we observed an increased macrophage infiltration in response to chemical injury in the central corneas of the *Mmp12^−/−^* mice compared to WT mice [15]. In the current study, we used flow cytometry to characterize the MP cells over the whole cornea (not only the central part), and despite initially observing a trend, the frequencies of MP subsets based on Ly6C expression did not reach statistical significance (*p* > 0.05) between MMP12 knockout and WT mice. Results from these two studies thus suggest MMP expression by monocytes as an important factor for their migration through the tissue matrix. In particular, MMP12 expression appears to inhibit monocyte migration through the cornea stroma. We previously showed that compared to WT mice, myeloid cells from *Mmp12^−/−^* mice are less dynamic, show a decreased velocity, and have only a small increase in track lengths and displacement after corneal injury [15,16]. Because MP cells are not evenly distributed over the cornea [25,26], the role of different MP populations in corneal wound healing may depend on how far and where these cells migrate into the cornea.

The corneas from MMP12 knockout mice presented a statistically significant reduction in the frequency of CD64^+^ macrophages on day 6 compared to days 1 and 3 (*p* = 0.0196; *p* = 0.0438) and a statistically significant (*p* = 0.0061) increase in the frequency of dendritic cells on day 6 post chemical injury compared to day 1, while the trend of progressive increase in dendritic cells observed in wild-type corneas did not reach statistical significance (*p* = 0.1982). Dendritic cells are antigen-presenting cells considered to be part of the MP system. This result may be seen as complementary to the observation of a progressive increase in the frequency of dendritic cells, reaching statistical significance after 7 days, in a model of dry eye and ocular surface inflammation [27].

Using a model of corneal epithelial and anterior stroma scraping, Sahu et al. investigated the kinetics of inflammatory cell recruitment over the initial 12 h after injury [28]. They found that the frequency of mast cells progressively increased over the initial 6 h, before declining to baseline at 12 h after injury, while neutrophil infiltration of the cornea progressively increased during the 12 h follow-up after injury. In our study, we focused on later time points and on the kinetics of the mononuclear phagocytes, but we did notice the presence of a population of other immune cells (CD45^+^CD11b^+^ F4/80^−^) that includes neutrophils, that was not present in the uninjured corneas but was elevated at days 1 and 3 after injury. These findings complement the observations reported by Sahu et al. and may suggest that the frequency of neutrophils remains elevated for at least 3 days but not until day 6 post-corneal injury (Figure 2). Neutrophils are involved in the early phase of the immune response and can be mediators of collateral tissue damage [29].

The early immune response of the cornea has been also studied using flow cytometry by Liu et al. [18]. After producing a 2 mm corneal epithelial injury, they followed the mouse wounds for up to 36 h. Unlike the isolated epithelial injury model, the chemical corneal injury model used in this study is a well-established model for studying corneal fibrosis [30], a problem that can lead to visual impairment and blindness. The NaOH corneal injury model affects the epithelial and stromal layers and induces a robust increase in MMP expression, inflammatory cytokine release, and intense corneal immune response [31]. Using this model, we previously found that F4/80^+^ cells are heavily recruited during the first week after injury and that the loss of MMP12 resulted in a higher number of recruited F4/80^+^ cells at 6 days post-injury and increased corneal fibrosis at 3 weeks post-injury [15]. This study provides a more detailed analysis of the MP subsets in the acute response to chemical injury. The Ly6C^high^ (proinflammatory) subset was predominant on day 1 post-injury for both WT and *Mmp12^−/−^* mice, and trended higher in *Mmp12^−/−^* mice compared with WT mice on days 1 and 6 post-injury (Figure 3). Additionally, a significant increase in CD11c^+^ dendritic cells was observed on day 6 post-injury in the *Mmp12^−/−^* mice. This higher expression of Ly6C^high^ cells and CD11c^+^ dendritic cells during the first 6 days after injury in *Mmp12^−/−^* mice compared with WT mice may contribute to the higher levels of corneal fibrosis we previously observed in the *Mmp12^−/−^* mice compared with WT mice at 3 weeks post-injury [15]. Interestingly, in a mouse model of cardiac fibrosis, the depletion of bone marrow-derived CD11c^+^ cells significantly reduced ventricular fibrosis [32] and suggests that therapies targeting these cells may have the potential for preventing tissue fibrosis.

Leukocytes that are not part of the MP system were not the focus of this study; therefore, specific markers for cells such as neutrophils, natural killers, and mast cells were not added to our antibody panel.

Our study builds on previous work by using well-known cell markers to characterize the phenotype and dynamic of MP cells over a period of 6 days after corneal injury. Our results show an early increase in the frequency of monocytes and Ly6C^high^ cells, followed later by an increase in Ly6C^int^ cells, Ly6C^neg^ cells, and CD11c^+^ dendritic cells. These findings provide an insight into the kinetics and functional roles of inflammatory and restorative MPs following corneal injury. Further analyses of these distinct MP populations using lineage tracing systems and single-cell transcriptomics may guide the development of MP-targeted therapies.

## 4. Materials and Methods

### 4.1. Animals

Breeding colonies of mice homozygous for the null allele of the Mmp12 [33] and wild-type mice on an FVB/n background were maintained under pathogen-free conditions in the UCSF barrier facility. Experiments were performed with littermate 8- to 12-week-old male and female mice. All animal protocols were approved by the UCSF Institutional Animal Care and Use Committee and all procedures were performed in accordance with the Association for Research in Vision and Ophthalmology Statement for the Use of Animals in Ophthalmic and Vision Research.

### 4.2. Corneal Chemical Injury

Corneal alkali burn injuries were performed on mice as previously described [34]. Briefly, mice were anesthetized by isoflurane inhalation (Baxter Pharmaceutical, Deerfield, IL) and by topical application of 0.5% Proparacaine (Akorn Inc., Buffalo Grove, IL, USA) placed on the cornea before and after the chemical burn. An alkaline burn was created by applying filter paper 2.5 mm in diameter soaked in 0.1 N NaOH (Sigma, St. Louis, MO, USA) for 30 s to the central cornea followed by rinsing with 1 mL of phosphate-buffered saline (PBS).

### 4.3. Flow Cytometry Analysis

Unwounded (control) and wounded whole globes were collected at 1, 3, and 6 days after injury. Corneas were removed and 4 corneas from 2 mice were combined into a single sample. The pooled corneas were cut into small pieces and digested with collagenase type I (1640 units in 400 μL; C0130 Sigma Aldrich, St. Louis, MO, USA) for 1.5 h at 37 °C. Cells were then washed with FACS buffer (2% fetal bovine serum in PBS) and incubated for 30 min in a FACS buffer solution containing CD16/32 Fc block (1:1000; UCSF monoclonal antibody core). Cells were then washed again with FACS buffer and stained for 30 min with a cocktail of the following antibodies: CD45-FITC, CD11B-Pacific Blue, F4/80-APC, Ly-6C-PerCP/Cy5, CD64-PE-Dazzle, CD11c-BrilliantViolet 605, and Zombie-Aqua Viability Dye (Biolegend, San Diego, CA, USA). Cells were washed and then fixed with 2% PFA in PBS overnight (at 4 °C). Cell profiles were acquired on an LSRII flow cytometer (Becton Dickinson, Franklin Lakes, NJ, USA), configured with the lasers 405 nm, 488 nm, 532 nm, and 633 nm and used for each fluorochrome as excitation-emission: Pacific Blue 405-BP450/50, Zombie-Aqua 405-BP525/50, BV605 405-BP605/12, FITC 488-BP525/50, PerCp/Cy5 488-685/35, PE-Dazzle 532-BP610/20, APC 633-BP660/20.

Post-acquisition analysis was performed using FCS Express 7 software (De Novo Software, Glendale, CA, USA), gating was based on unstained and fluorescence minus one controls (Supplementary Figure S1).

### 4.4. Statistical Analysis

The average percentages of each cell population were obtained using FCS Express 7 software. All data are expressed as the mean ± SEM. The effects of time and gene knockout on the cell population’s phenotype were evaluated using two-way ANOVA followed by Tukey *post hoc* test for multiple comparisons. Statistical comparisons were made using Prism 8.3 statistical software (GraphPad, San Diego, CA, USA). An alpha level of *P* less than or equal to 0.05 was chosen as the criterion of significance.

## Data Availability

The data presented in this study are available from the corresponding author upon reasonable request.

## Supplementary Materials

The following supporting information can be downloaded at: https://www.mdpi.com/article/10.3390/ijms23052574/s1.
