# Supplementary figures and images for: Characterization of Recruited Mononuclear Phagocytes following Corneal Chemical Injury
Source: Int J Mol Sci. 2022 Feb 25;23(5):2574. doi: 10.3390/ijms23052574 (PMC8910730; doi:10.3390/ijms23052574)

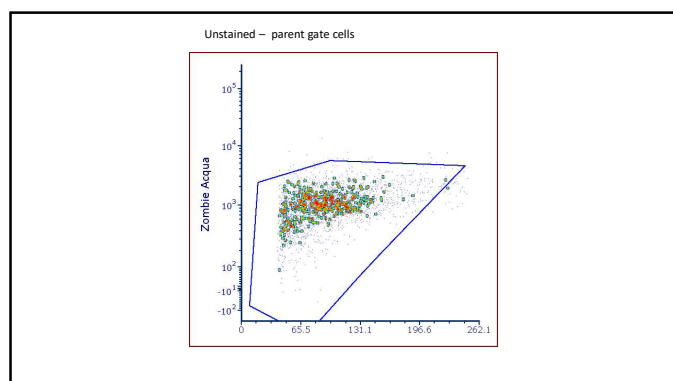

1

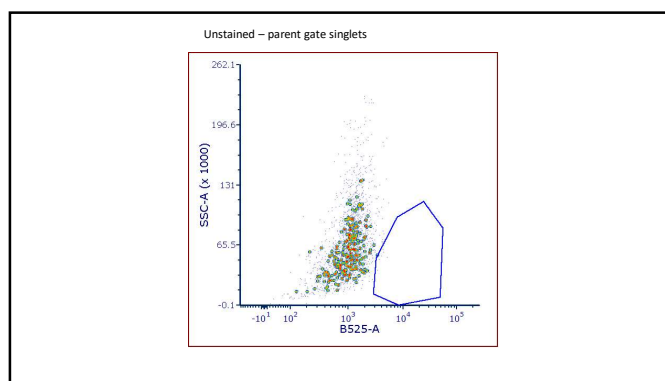

2

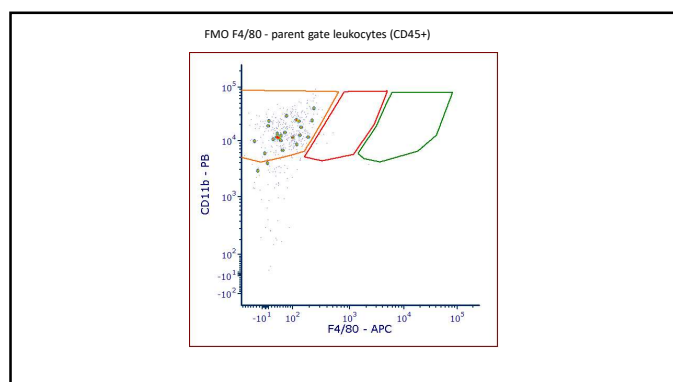

3

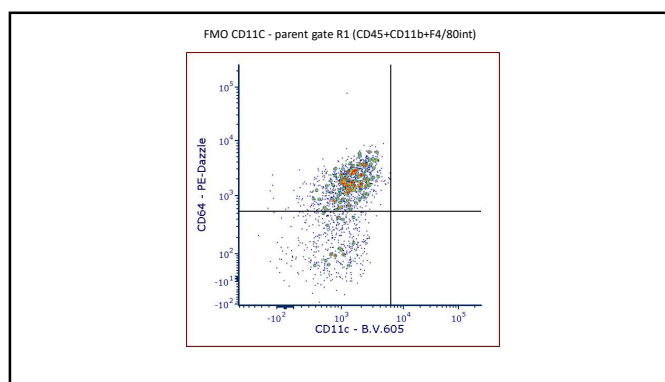

4

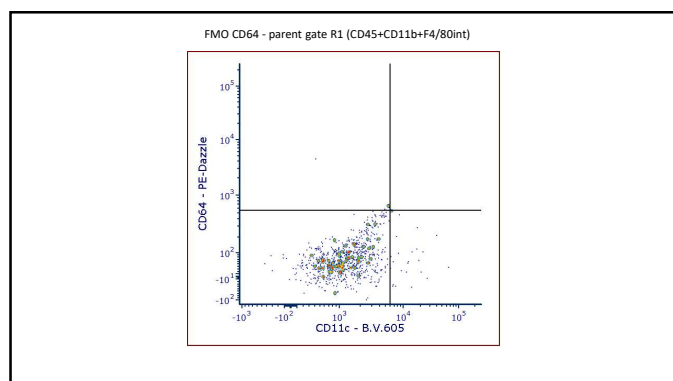

5

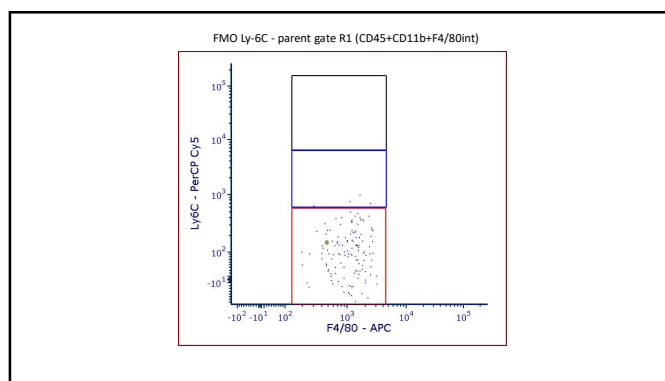

6

Supplement: Supplementary file 1 [file ijms-23-02574-s001.zip › ijms-1602513-supplementary.pdf]
